# Opportunities to Prevent Overdose Deaths Involving Prescription and Illicit Opioids, 11 States, July 2016–June 2017

**DOI:** 10.15585/mmwr.mm6734a2

**Published:** 2018-08-31

**Authors:** Christine L. Mattson, Julie O’Donnell, Mbabazi Kariisa, Puja Seth, Lawrence Scholl, R. Matthew Gladden

**Affiliations:** ^1^Division of Unintentional Injury Prevention, National Center for Injury Prevention and Control, CDC; ^2^Oak Ridge Institute for Science and Education, Oak Ridge, Tennessee.

## Abstract

In 2016, 63,632 drug overdose deaths occurred in the United States, 42,249 (66.4%) of which involved opioids (*1*). The development of prevention programs are hampered by a lack of timely data on specific substances contributing to and circumstances associated with fatal overdoses. This report describes opioid overdose deaths (referred to as opioid deaths) for decedents testing positive for prescription opioids (e.g., oxycodone and hydrocodone), illicit opioids (e.g., heroin, illicitly manufactured fentanyl, and fentanyl analogs), or both prescription and illicit opioids, and describes circumstances surrounding the overdoses, in 11 states participating in CDC’s Enhanced State Opioid Overdose Surveillance (ESOOS) program.* During July 2016–June 2017, among 11,884 opioid overdose deaths, 17.4% of decedents tested positive for prescription opioids only, 58.7% for illicit opioids only, and 18.5% for both prescription and illicit opioids (type of opioid could not be classified in 649 [5.5%] deaths). Approximately one in 10 decedents had been released from an institutional setting in the month preceding the fatal overdose. Bystanders were reportedly present in approximately 40% of deaths; however, naloxone was rarely administered by a layperson. Enhanced surveillance data from 11 states provided more complete information on the substances involved in and circumstances surrounding opioid overdose deaths. Consistent with other emerging evidence and recommendations,^†^ these data suggest prevention efforts should prioritize naloxone distribution to persons misusing opioids or using high dosage prescription opioids and to their family members and friends. In addition, these data suggest a need to expand treatment and support for persons who have experienced a nonfatal overdose and to expand treatment in detention facilities and upon release.

CDC funds 32 states and the District of Columbia to abstract death certificate and medical examiner and coroner data, including toxicology results, on opioid deaths, through the State Unintentional Drug Overdose Reporting System, a component of ESOOS. Data are collected on death scene investigations (e.g., evidence of illicit or prescription drug use), circumstances occurring close in time to the death (e.g., presence of bystanders), history of substance use treatment, prior history of overdose, and demographics. For all opioid deaths classified as unintentional or of undetermined intent, states list all positive tests for opioid and nonopioid substances (“present” or “detected”), and whether the medical examiner or coroner determined that the substance contributed to the overdose death (“involved”).^§^ CDC analyzed demographics, routes of administration, co-use of other substances, and overdose circumstances by involvement of prescription opioids only,^¶^ illicit opioids only,** or the presence of both prescription and illicit opioids,^††^ for deaths that occurred during July 2016–June 2017 in 11 ESOOS states.^§§^

Among 11,884 opioid deaths, 2,066 (17.4%) involved prescription opioids only, 6,975 (58.7%) involved illicit opioids only, and for 2,194 (18.5%) both prescription and illicit opioids were detected; type of opioid could not be classified in 649 (5.5%) deaths, leaving 11,235 deaths for analysis. Among deaths for which both prescription and illicit opioids were detected, medical examiners or coroners determined that both prescription and illicit opioids contributed to 59.2% of deaths, illicit opioids alone contributed to 39.8% of deaths, and prescription opioids alone contributed to 1.0% of deaths. The percentage of deaths involving different opioid types varied across states (Figure), with the highest percentages of prescription opioid–only deaths in the West (Oklahoma: 72.2%; New Mexico: 35.0%), and the highest percentages of illicit opioid–only deaths, ranging from 47.6% to 72.1%, in the Northeast (Maine, Massachusetts, New Hampshire, and Rhode Island) and the Midwest (Missouri, Ohio, West Virginia, and Wisconsin). Kentucky had the highest percentage of deaths with both prescription and illicit opioids (26.5%) present, followed by Missouri (25.1%).

**FIGURE:**
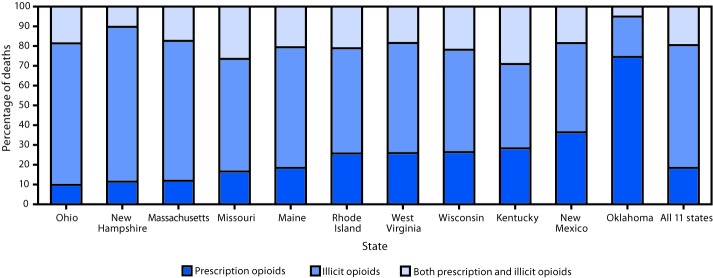
Percentage of opioid overdose deaths in which prescription opioids only,* illicit opioids only,**^†^** or both prescription and illicit opioids**^§^** were detected, by state — 11 states, July 1, 2016–June 30, 2017 * Oxycodone, oxymorphone, hydrocodone, hydromorphone, tramadol, buprenorphine, methadone, meperidine, tapentadol, dextrorphan, levorphanol, propoxyphene, noscapine, pentazocine, and phenacetin. Brand names (e.g., Opana), metabolites (e.g., nortramadol) of these substances, and these substances in combination with nonopioids (e.g., acetaminophen-oxycodone) were included as prescription opioids. Morphine and codeine were coded as prescription opioids if scene or other evidence indicated their presence as a result of consumption of prescription morphine or codeine, rather than as a result of metabolism of or impurities of heroin, respectively. Fentanyl was coded as a prescription opioid if scene or other evidence indicated likely consumption of prescription fentanyl rather than illicitly manufactured fentanyl. Decedents might have tested positive for other nonopioid substances. This analysis does not distinguish whether prescription drugs were prescribed to the decedent or diverted. ^†^ Heroin, fentanyl analogs, and U-47700. Fentanyl was coded as illicit if scene or other evidence indicated that it was more likely illicitly manufactured than pharmaceutical. Decedents might have tested positive for other nonopioid substances. ^§^ Deaths were coded as positive for both prescription and illicit opioids if one or more opioids from both categories were detected on postmortem toxicology testing. Decedents might have tested positive for other nonopioid substances.

Among prescription opioid–only deaths, the median age of decedents was 47 years, 51.0% were female, and 86.2% were non-Hispanic white (white). Among illicit opioid–only deaths, the median age of decedents was 36 years, 73.0% were male, and 81.1% were white. Among deaths for which both prescription and illicit opioids were detected, decedents’ median age was 39 years, 70.5% were male, and 83.6% were white (Table).

**TABLE:** Demographic characteristics of persons who died from opioid overdose and overdose circumstance factors by substance type — 11 states, July 1, 2016─June 30, 2017

Characteristic	Type of opioid(s) detected		
	Prescription opioids only N = 2,066 (17.4%)	Illicit opioids only N = 6,975 (58.7%)	Prescription and illicit opioids N = 2,194 (18.5%)
	No. (%)	No. (%)	No. (%)
Type of opioid(s) contributing to death*			
Prescription opioids only	2,066 (100.0)	—**^†^**	22 (1.0)
Illicit opioids only	—**^†^**	6,975 (100.0)	873 (39.8)
Both illicit and prescription opioids	—**^†^**	—**^†^**	1,299 (59.2)
**Age group (yrs)** ^§^			
15–24	79 (3.8)	714 (10.2)	130 (5.9)
25–34	307 (14.9)	2,346 (33.6)	608 (27.7)
35–44	500 (24.2)	1,825 (26.2)	635 (28.9)
45–54	621 (30.1)	1,298 (18.6)	485 (22.1)
55–64	456 (22.1)	708 (10.2)	291 (13.3)
≥65	103 (5.0)	84 (1.2)	45 (2.1)
Median age (interquartile range) in years^¶^	47 (37–55)	36 (29–47)	39 (32–50)
**Sex** ^§^			
Male	1,013 (49.0)	5,089 (73.0)	1,546 (70.5)
Female	1,053 (51.0)	1,886 (27.0)	648 (29.5)
**Race and Hispanic origin** ^§^			
White, non-Hispanic	1,780 (86.2)	5,657 (81.1)	1,833 (83.6)
Black, non-Hispanic	100 (4.8)	685 (9.8)	206 (9.4)
Other, non-Hispanic	51 (2.5)	88 (1.3)	16 (0.7)
Hispanic	100 (4.8)	421 (6.0)	112 (5.1)
**Route of administration**			
Evidence of injection^§^	136 (6.6)	3,428 (49.2)	958 (43.7)
No evidence of injection; evidence of other route^§^	783 (37.9)	1,194 (17.1)	382 (17.4)
Evidence of snorting^§^	85 (10.9)	743 (62.2)	210 (55.0)
Evidence of ingestion^§^	669 (85.4)	416 (34.8)	211 (55.2)
Evidence of smoking^§^	34 (4.3)	251 (21.0)	54 (14.1)
Evidence of transdermal	65 (8.3)	—**^†^**	—**^†^**
No evidence of route^§^	1,147 (55.5)	2,353 (33.7)	854 (38.9)
**Other substance(s) detected**			
Cocaine^§,^**	207 (10.0)	2,432 (34.9)	763 (34.8)
Methamphetamines^§^	155 (7.5)	737 (10.6)	277 (12.6)
Benzodiazepines^§^	1,065 (51.6)	1,676 (24.0)	976 (44.5)
Gabapentin^§^	447 (21.6)	564 (8.1)	326 (14.9)
**Released from an institution 1 month before death**			
Released from any institutional setting^§^	140 (6.8)	726 (10.4)	200 (9.1)
Released from jail, prison, or detention facility^§^	22 (1.1)	343 (4.9)	67 (3.1)
Released from residential alcohol or substance use treatment facility^§^	22 (1.1)	216 (3.2)	53 (2.5)
Released from a hospital^§^	81 (4.1)	107 (1.6)	54 (2.6)
**Previous drug overdose** ^§^	193 (9.3)	1,053 (15.1)	297 (13.5)
**Died when bystander was present**	860 (41.6)	3,072 (44.0)	987 (45.0)
**Naloxone administered by layperson** ^§,^ **^††^**	8 (0.8)	169 (4.3)	52 (4.4)

* By definition, for the categories of “only prescription opioids” and “only illicit opioids” detected, the contributing opioid type could only be “prescription opioids only” or “illicit opioids only,” respectively. For the category of “both prescription and illicit opioids,” either prescription opioids only, illicit opioids only, or both could have contributed to death. Decedents might have tested positive for other nonopioid substances. This analysis does not distinguish between prescription drugs prescribed to the decedent and those that were diverted.

**^†^** Data suppressed because of <5 deaths, or suppressed to prohibit calculation of other suppressed cell.

^§^ Indicates overall chi-squared test statistic has a p-value <0.001.

^¶^ Statistically significant differences in mean age (p-value <0.001) were identified for prescription opioid deaths (46.3 years), illicit opioid deaths (38.4 years), and prescription and illicit (40.9 years).

** Indicates the presence of cocaine or a cocaine metabolite.

**^††^** Among deaths for which naloxone administration status was known: 1,032 for prescription opioids only, 3,927 for illicit opioids only, and 1,173 for prescription and illicit opioids.

Evidence of injection drug use was found in approximately half of illicit opioid deaths, but only 6.6% of prescription opioid–only deaths. Other drugs were frequently detected in opioid deaths (Table). Benzodiazepines and gabapentin were detected in 51.6% and 21.6% of prescription opioid–only deaths, respectively. Among illicit opioid–only deaths, cocaine was detected in 34.9% of deaths and benzodiazepines were detected in 24.0% of deaths. Among deaths for which both prescription and illicit opioids were detected, benzodiazepines were detected in 44.5% and cocaine in 34.8%.

Approximately one in 10 decedents had evidence of having been released from an institutional setting in the month preceding the fatal overdose, with the most common settings being jail, prison, or detention facilities when only illicit opioids were involved (4.9%), and hospitals when only prescription opioids were involved (4.1%). Previous drug overdose at any time before the fatal overdose was noted in 15.1% of illicit opioid–only deaths, 13.5% of deaths with both prescription and illicit opioids present, and 9.3% of prescription opioid–only deaths. Bystanders were reported to have been present in 44% of opioid deaths, but naloxone was seldom administered by a layperson (in approximately 4% of deaths involving only illicit opioids and 0.8% of prescription opioid–only deaths).

## Discussion

This report is one of the first to use medical examiner and coroner reports across multiple states and provides information that can be used to better inform prevention and response programs related to opioid overdose deaths. Specifically, among these 11 states, there is improved understanding of prescription and illicit opioid involvement, polysubstance use, and potential missed opportunities to intervene to prevent opioid overdose deaths. Previous efforts to differentiate illicit and prescription opioid deaths were limited by grouping within the same drug categories (e.g., synthetic opioids, excluding methadone) and by the difficulty in determining whether detection of morphine or fentanyl by forensic toxicology testing indicates the presence of prescription or illicit opioids (*2*,*3*).^¶¶^ Findings from this analysis indicate that illicit opioids were a major driver of opioid deaths, especially among younger persons, and were detected in approximately three of four deaths overall. Prescription opioids were detected in approximately four of 10 deaths. Illicit opioids predominated in all states except Oklahoma.

Among these 11 states, the evolving opioid overdose epidemic differentially affects states and regions, but most states were simultaneously struggling with a complex mix of prescription and illicit opioid deaths. In this analysis, four polysubstance use patterns highlight an urgent need for targeted and comprehensive action. First, approximately half of prescription opioid–only deaths tested positive for benzodiazepines, which are known to depress the central nervous system and increase the risk of overdose and death.*** This high-risk drug-use pattern can be targeted for intervention. Second, gabapentin (a nonopioid medication commonly prescribed for neuropathic pain), was found in approximately one in five prescription opioid–only deaths and in approximately one in 10 deaths in the other groups. Consistent with recent reports (*4*), the combined use of gabapentin and opioids might be an indicator of high-risk opioid misuse and requires further study. In the illicit opioid–only group, the percentage of deaths testing positive for cocaine and methamphetamine is similar to other reports (*5*). Finally, extensive use of cocaine and benzodiazepines among deaths where both prescription and illicit opioids were detected highlights the need for prevention and treatment programs to address polysubstance use (*6*).

Identification of circumstances surrounding overdose deaths can help inform prevention programs and efforts to target resources. Approximately one in 10 decedents had been released from an institution in the month before the fatal overdose. Rhode Island found that expanding enrollment in a medication-assisted treatment program for incarcerated persons was associated with a 60% decrease in postincarceration overdose deaths (*7*). For the 14% of decedents with previous overdoses, there might have been opportunities for linkage to care and treatment services, especially if the overdose involved an emergency department visit (*8*). The proportions of decedents with evidence of recent release from an institution and of a previous overdose were higher among deaths involving illicit opioids. Similar to earlier findings (*9*), approximately half of the decedents overdosed when bystanders were present. Although distribution of naloxone to laypersons has rapidly expanded and been determined to be effective,^†††^ broader distribution and education about overdose signs and symptoms are needed.

The findings in this report are subject to at least five limitations. First, because there is no national standard for forensic toxicology testing, testing protocols vary across jurisdictions, which affects whether substances were detected. Second, jurisdictions vary in how they classify whether substances with positive toxicology results contribute to death. Third, evidence of overdose-specific circumstances should be interpreted with caution because it relies upon availability of information within medical examiner and coroner reports and focuses on information from a period close to death; thus, prevalence of circumstances is likely underestimated. Fourth, missing information might have resulted in some misclassification of prescription and illicit substance use; however, this was minimized by using detailed toxicology results and scene evidence. Finally, the results are limited to the 11 participating states and cannot be generalized to the United States.

Among 11 states, illicit opioids were a major driver of opioid overdose deaths; however, prescription opioids also contribute to a substantial number of these deaths. Interventions should be guided by the substances detected and contributing to overdose deaths in a given locale and might differ for overdoses involving prescription or illicit opioids, or both. For example, for preventing illicit opioid overdose, integrating public health strategies within public safety (e.g., law enforcement providing linkages to care for persons with substance use disorders), as well as using syringe services programs for naloxone distribution, providing access to treatment, and addressing blood borne infections might have a larger impact. To prevent prescription opioid overdose, strategies might emphasize prescription drug monitoring programs, face-to-face education of prescribers by trained health care professionals, typically pharmacists, physicians, or nurses (a process known as academic detailing), and implementation of the CDC Guideline for Prescribing Opioids for Chronic Pain (*10*). However, interventions should not focus exclusively on one opioid type because the epidemic continues to evolve, and use of opioids along with other substances is common. Continued and increased attention should capitalize on opportunities for overdose prevention including linking to treatment during and upon release from an institution or after a nonfatal overdose and expanding naloxone access.

**Summary**

**What is already known about this topic?**

In 2016, opioids were involved in 42,249 U.S. overdose deaths.

**What is added by this report?**

Among 11 reporting states, most (58.7%) opioid overdose deaths involved illicit opioids only, followed by those where both illicit and prescription opioids were detected (18.5%); 17.4% of deaths involved prescription opioids only. Bystanders to the overdose, who could potentially intervene, were documented in 44% of deaths; however, laypersons rarely administered naloxone.

**What are the implications for public health practice?**

Development of overdose prevention programs should consider the types of opioids contributing to deaths, link persons to treatment during and upon release from an institution or after a nonfatal overdose, and expand naloxone distribution to laypersons.

## Footnotes

* CDC’s Enhanced State Opioid Overdose Surveillance program funded 12 states through a competitive application process in fiscal year 2016, and an additional 20 states and the District of Columbia in fiscal year 2017. Data were available for Kentucky, Maine, Massachusetts, Missouri, New Hampshire, New Mexico, Ohio, Oklahoma, Rhode Island, West Virginia, and Wisconsin for this report. https://www.cdc.gov/drugoverdose/foa/state-opioid-mm.html.

† https://www.surgeongeneral.gov/priorities/opioid-overdose-prevention/naloxone-advisory.html.

§ State Unintentional Drug Overdose Reporting System (SUDORS) estimates of opioid-involved overdose deaths might differ from those of the National Vital Statistics System because SUDORS uses preliminary death certificate data and collects additional information from medical examiner and coroner reports, which are abstracted within 8 months of death. In SUDORS, an opioid-involved overdose death either was identified through review of the medical examiner/coroner report or had *International Classification of Diseases, Tenth Revision* (ICD-10) underlying cause-of-death codes X40–44 (unintentional) or Y10–Y14 (undetermined) and multiple cause-of-death codes of T40.0, T40.1, T40.2, T40.3, T40.4, or T40.6 on the death certificate. Data for this report were downloaded on June 30, 2018, and might differ from reports using earlier data.

¶ Substances coded as prescription opioids were oxycodone, oxymorphone, hydrocodone, hydromorphone, tramadol, buprenorphine, methadone, meperidine, tapentadol, dextrorphan, levorphanol, propoxyphene, noscapine, pentazocine, and phenacetin. Brand names (e.g., Opana), metabolites (e.g., nortramadol) of these substances, and these substances in combination with nonopioids (e.g., acetaminophen-oxycodone) were included as prescription opioids as well. Morphine and codeine were coded as prescription opioids if scene or other evidence indicated their presence as a result of consumption of prescription morphine or codeine, rather than as a result of metabolism of or impurities of heroin, respectively. Fentanyl was coded as a prescription opioid if scene or other evidence indicated likely consumption of prescription fentanyl rather than illicitly manufactured fentanyl. Decedents might have tested positive for other nonopioid substances. This analysis does not distinguish between prescription drugs prescribed to the decedent and those that were diverted.

** Substances coded as illicit opioids were heroin, fentanyl analogs, and U-47700. Fentanyl was coded as illicit if scene or other evidence indicated that it was more likely to have been illicitly manufactured than pharmaceutical. Decedents might have tested positive for other nonopioid substances.

†† Deaths were coded as positive for both prescription and illicit opioids if one or more opioids from both categories were detected on postmortem toxicology testing. Decedents might have tested positive for other nonopioid substances.

§§ Twelve states were funded through a competitive application process in September 2016 (Kentucky, Maine, Massachusetts, Missouri, New Hampshire, New Mexico, Ohio, Oklahoma, Pennsylvania, Rhode Island, West Virginia, and Wisconsin). An additional 20 states (Alaska, California, Connecticut, Delaware, Florida, Georgia, Illinois, Indiana, Louisiana, Maryland, Michigan, Minnesota, Nevada, New Jersey, North Carolina, Tennessee, Utah, Vermont, Virginia, and Washington) and the District of Columbia were funded in September 2017. Data were available for 11 of the 33 jurisdictions for this report.

¶¶
http://ajph.aphapublications.org/doi/10.2105/AJPH.2017.304265 .

*** The Food and Drug Administration issued its strongest warning against combining benzodiazepines with opioids because of risk for overdose. https://www.fda.gov/Drugs/DrugSafety/ucm518473.htm.

†††
https://www.bmj.com/content/346/bmj.f174 .
